# How salicylic acid takes transcriptional control over jasmonic acid signaling

**DOI:** 10.3389/fpls.2015.00170

**Published:** 2015-03-25

**Authors:** Lotte Caarls, Corné M. J. Pieterse, Saskia C. M. Van Wees

**Affiliations:** Plant-Microbe Interactions, Department of Biology, Faculty of Science, Utrecht UniversityUtrecht, Netherlands

**Keywords:** hormone crosstalk, transcription factors, regulation of gene expression, plant immunity, post-translational modifications

## Abstract

Transcriptional regulation is a central process in plant immunity. The induction or repression of defense genes is orchestrated by signaling networks that are directed by plant hormones of which salicylic acid (SA) and jasmonic acid (JA) are the major players. Extensive cross-communication between the hormone signaling pathways allows for fine tuning of transcriptional programs, determining resistance to invaders and trade-offs with plant development. Here, we give an overview of how SA can control transcriptional reprogramming of JA-induced genes in *Arabidopsis thaliana*. SA can influence activity and/or localization of transcriptional regulators by post-translational modifications of transcription factors and co-regulators. SA-induced redox changes, mediated by thioredoxins and glutaredoxins, modify transcriptional regulators that are involved in suppression of JA-dependent genes, such as NPR1 and TGA transcription factors, which affects their localization or DNA binding activity. Furthermore, SA can mediate sequestering of JA-responsive transcription factors away from their target genes by stalling them in the cytosol or in complexes with repressor proteins in the nucleus. SA also affects JA-induced transcription by inducing degradation of transcription factors with an activating role in JA signaling, as was shown for the ERF transcription factor ORA59. Additionally, SA can induce negative regulators, among which WRKY transcription factors, that can directly or indirectly inhibit JA-responsive gene expression. Finally, at the DNA level, modification of histones by SA-dependent factors can result in repression of JA-responsive genes. These diverse and complex regulatory mechanisms affect important signaling hubs in the integration of hormone signaling networks. Some pathogens have evolved effectors that highjack hormone crosstalk mechanisms for their own good, which are described in this review as well.

## Introduction

The activation of inducible immune responses in the plant is tightly regulated, ensuring an effective and cost-efficient response to pathogenic microbes and herbivorous insects (Vos et al., 2013a). Recognition of an attacker leads to accumulation of signaling molecules like the plant hormones salicylic acid (SA) and jasmonic acid (JA) and its derivatives, which play major roles in the activation of downstream defense responses (reviewed by Pieterse et al., 2012). Generally speaking, SA activates resistance against biotrophic pathogens, while JA is critical for activation of defense against herbivorous insects and necrotrophic pathogens. The SA- and JA-responsive signaling pathways are interdependent and act in complex networks. Other hormones participate in these defense signaling networks as well and can consequently modulate the outcome of the activated defense arsenal. Abscisic acid (ABA) and ethylene can act synergistically with distinct JA-regulated responses, while they generally antagonize SA responses. Auxin, gibberellins, and cytokinins can repress defense-related processes to prioritize growth of the plant, and vice versa their action can be suppressed by SA or JA leading to activation of defense at the expense of plant growth (Pieterse et al., 2012).

Most knowledge on hormone signaling pathways stems from work on the molecular genetic model plant *Arabidopsis thaliana.* Consequently, this review is based primarily on research with *Arabidopsis*, but we are aware that other plant species may regulate the interplay between hormone signaling pathways differently. We aim to focus on general mechanisms affecting transcriptional regulation that could also apply to other plant species. Hormone-modulated regulation of disease resistance is primarily achieved through effects on gene transcription. Activation or repression of target genes is accomplished by physical interaction between *trans*-acting proteins, such as transcription factors, and *cis*-acting DNA elements. Transcription factors and co-regulators can themselves be controlled at the transcriptional level, but they are also subject to post-translational modification through reduction or oxidation, sequestration, phosphorylation, degradation, or interaction with other transcription factors or co-factors (Moore et al., 2011). Moreover, transcriptional activation is determined by the accessibility of *cis*-acting elements, which can be influenced by remodeling of chromatin through modifications of histones (Liu et al., 2014).

**FIGURE 1:**
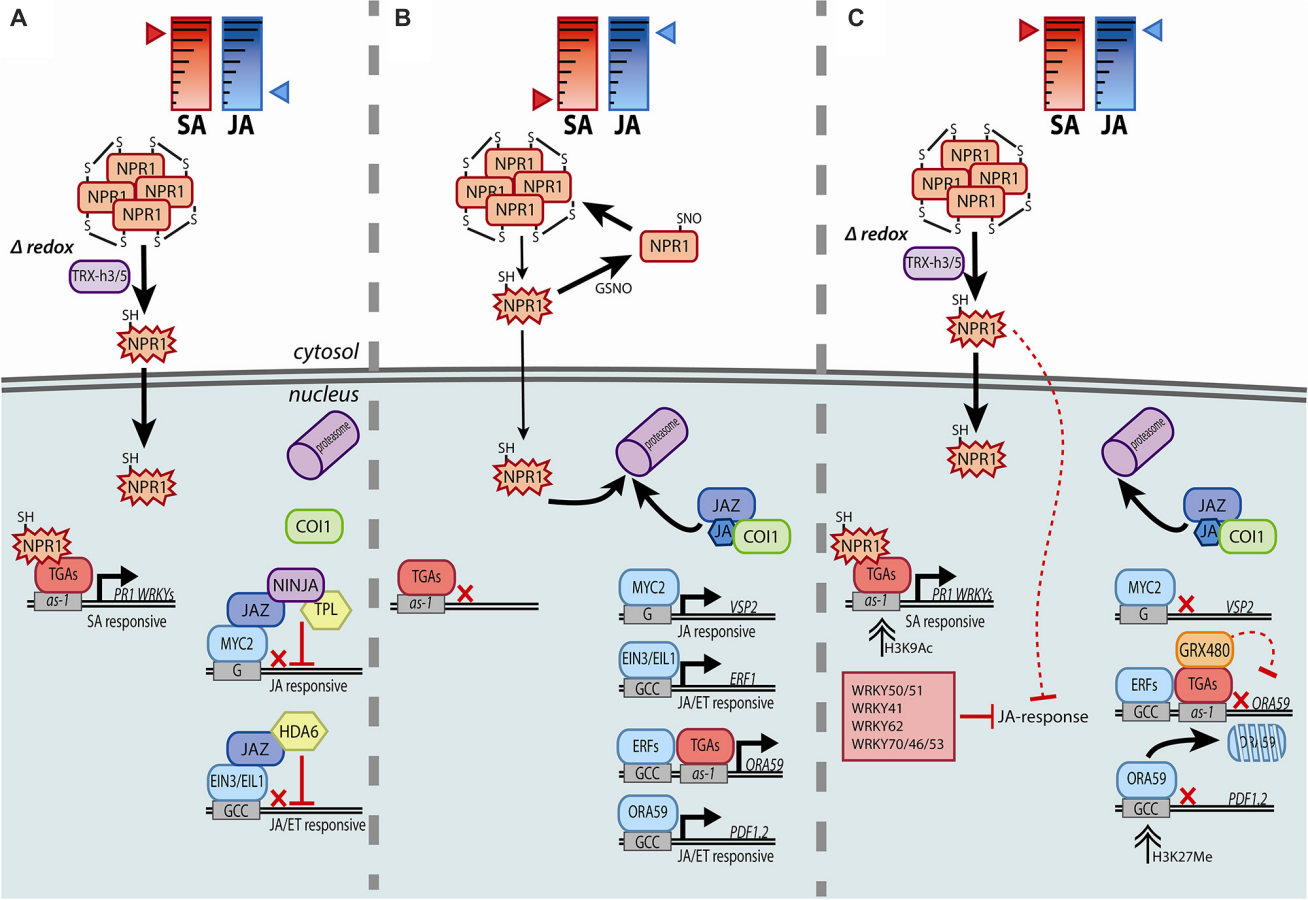
**Simplified model of the molecular machinery involved in the transcriptional regulation of the SA signaling pathway (A), the JA signaling pathway (B), or the antagonism of SA on the JA signaling pathway (C).** By inducing reduction and monomerization of NPR1, SA activates NPR1 (star-shaped), which then triggers gene expression in the nucleus. JA-responsive genes are kept in check by JAZ repressors in the absence of JA. In the presence of JA, MYC or ERF transcription factors activate JA-responsive genes, but only if SA is absent. Activation of both the SA and JA signaling pathways leads to antagonism of JA-responsive gene expression by SA. There are indications for roles in SA/JA crosstalk for cytosolic NPR1, and nuclear localized TGAs, GRX480, and WRKYs. See text for details on the molecular processes underlying the transcriptional control, like redox signaling, sequestration, degradation, phosphorylation, and chromatin modification. Solid lines indicate established (in)activities and dashed lines hypothesized (in)activities, where black arrows specify activation and red blocks suppression. Red crosses indicate that gene transcription is hampered.

Transcriptional and post-translational regulatory mechanisms are important in both SA- and JA-controlled signaling pathways. In the SA pathway, activity of NPR1, which was identified as a master transcriptional co-regulator of SA-dependent genes, is tightly regulated by several SA-dependent modifications (reviewed by Fu and Dong, 2013). SA induces a biphasic fluctuation in the cellular redox state that can be sensed by NPR1, which then switches from an oligomer to monomer form by reduction of intermolecular disulfide bonds. Thioredoxins TRX-h5 and TRX-h3 catalyze the formation of NPR1 monomers, which translocate to the nucleus (**Figure 1A**). Regulation of NPR1 monomer levels in the nucleus is also dependent on SA. NPR1 and NPR1-homologs NPR3 and NPR4 were described to be SA-receptors (Fu et al., 2012; Wu et al., 2012). NPR3 and NPR4 act as CUL3 ligase adapter proteins in proteasome-mediated degradation of NPR1. NPR3 and NPR4 differ in both their binding affinity for SA and binding capacity to NPR1, so that SA levels determine when NPR1 is targeted for degradation. When SA levels are low, NPR4 interacts with NPR1, leading to its degradation, and in this way untimely transcriptional activation in absence of SA is prevented. High SA levels facilitate binding between NPR1 and NPR3, again leading to removal of NPR1 (Fu et al., 2012). This degradation of NPR1 is thought to help activate programmed cell death, of which NPR1 is a negative regulator. When SA levels are intermediate, interaction between NPR1 and NPR3 is prevented, allowing NPR1 to accumulate and activate SA-dependent defenses. By interacting with transcription factors of the TGA family, NPR1 acts as a co-activator of SA-induced gene transcription, activating SA marker genes such as *PR1*, but also several *WRKY* transcription factor genes, which then fine-tune and amplify downstream transcriptional responses (Wang et al., 2006; Eulgem and Somssich, 2007).

Master regulators of the JA signaling pathway are the F-box protein COI1 and the JAZ repressor proteins. In the absence of JA, JAZ repressor proteins associate with the co-repressor TPL via the adapter protein NINJA, or with HDA6, thereby repressing various transcription factors, among which MYC2, EIN3, and EIL1 (**Figure 1A**; reviewed by Song et al., 2014). COI1 binds to JA-Ile, the bioactive form of JA, which leads to targeting of JAZ repressor proteins for degradation by the proteasome. The successive release of transcriptional activators then leads to activation of JA-responsive genes (**Figure 1B**). Two branches are distinguished in JA-dependent signaling: (i) MYC2 is the master regulator of the MYC branch, which is co-regulated by JA and ABA, activating downstream marker genes *VSP2* and *LOX2* (Lorenzo et al., 2004; Vos et al., 2013b), while (ii) EIN3, EIL1, and ERF transcription factors like ERF1 and ORA59 regulate the ERF branch, which is co-regulated by JA and ET, activating the downstream marker gene *PDF1.2* (Zhu et al., 2011; Pieterse et al., 2012; Wasternack and Hause, 2013).

Recent work indicates that suppression of the JA-responsive pathway by SA (hereafter also referred to as SA/JA crosstalk) is predominantly regulated at the level of gene transcription (Van der Does et al., 2013). First, SA/JA crosstalk proved to be independent of downregulation of JA biosynthesis itself, as the SA-mediated suppression of MeJA-induced *PDF1.2* was intact in the JA biosynthesis mutant *aos/dde2* (Leon-Reyes et al., 2010b). Using the JA-receptor mutant *coi1-1* ectopically expressing *ERF1* to constitutively express downstream JA-responsive genes, Van der Does et al. (2013) further demonstrated that SA can suppress ERF1-activated *PDF1.2* independently of COI1. Moreover, using GCC:GUS reporter lines, the GCC-box, which is a crucial *cis*-element in the regulation of *PDF1.2* expression, was shown to be sufficient for SA/JA crosstalk. This indicates that SA antagonizes JA signaling downstream of COI1, possibly by interfering with JA-regulated transcription factors. The ERF transcription factor ORA59 was then demonstrated to be degraded by SA. At the SA signaling side, using mutant *npr1-1*, master regulator NPR1 was previously shown to be essential for suppression of JA-responsive gene expression (Spoel et al., 2003). Further, several WRKY and TGA transcription factors have been shown to be important for SA/JA crosstalk (Pieterse et al., 2012; Gimenez-Ibanez and Solano, 2013). However, the ways by which these transcriptional regulators down-regulate JA signaling in the presence of SA are largely unknown. In this review, we discuss the regulatory mechanisms that SA employs to repress JA-regulated transcriptional activity. Where relevant, examples of how other hormones interfere with hormone-dependent transcriptional regulation will be given.

## SA-Mediated Effects on Activity or Localization of Transcription Factors

### SA-Induced Modification of Transcriptional Regulators via Redox Signaling

The activation of the immune response in plants is associated with rapid production of reactive oxygen intermediates (ROI) and increased levels of nitric oxide (NO). Redox-sensing small-molecule couples, such as reduced and oxidized glutathione, can limit damage from these redox active molecules. Moreover, these redox sensors transduce changes in ROI and NO levels into posttranslational modifications by reduction or oxidation of cysteine residues of transcriptional regulators, causing changes in transcriptional activity (Frederickson and Loake, 2014). Redox signaling is important in SA signaling and moreover, SA-induced redox changes are associated with the suppression of JA responses as well.

#### Role of Reduction of Transcriptional Regulators in SA Signaling

In SA signaling, master regulator NPR1 is subject to several redox-dependent modifications. It sequesters in the cytoplasm as an oligomer, formed by intermolecular disulfide bonds, which are facilitated by *S*-nitrosylation of cysteine residues via NO donor *S*-nitrosoglutathione (GSNO; **Figure 1B**). SA triggers cycles of cellular reduction and oxidation, measurable for example by enhanced total glutathione levels and a higher ratio of reduced to oxidized glutathione after SA treatment (Spoel and Loake, 2011). In response to activation of the SA pathway, thioredoxins catalyze the reduction of intermolecular disulphide bonds, causing a conformational change of NPR1 to its monomeric form. As a monomer, NPR1 is able to translocate from the cytosol to the nucleus and activate downstream signaling (**Figure 1A**) (Mou et al., 2003; Koornneef et al., 2008a; Tada et al., 2008). Other transcriptional regulators functioning in the SA pathway are also redox controlled. Transcription factor TGA1 contains intramolecular disulfide bonds that prevent its interaction with NPR1. Only after reduction of these bonds under high SA conditions, TGA1 is able to interact with NPR1. Further *S*-nitrosylation and *S*-glutathionylation of the cysteine residues of TGA1 result in enhanced binding to DNA and activation of transcription (**Figure 1A**; Després et al., 2003; Lindermayr et al., 2010).

#### Role of the Redox State in SA/JA Crosstalk Signaling

Redox-mediated reduction of transcriptional regulators is not only essential for SA signaling, but is also implicated in SA/JA crosstalk. The enhancement in glutathione levels after SA treatment was shown to coincide exactly with the window of opportunity in which SA could suppress JA-induced *PDF1.2* expression, i.e., within 30 h after application of SA. In addition, treatment with glutathione synthesis inhibitor BSO blocked SA-mediated antagonism of *PDF1.2* expression (Koornneef et al., 2008a). Interestingly, JA can also influence the redox state of cells, but, in contrast to SA, it decreases the total amount of glutathione, and shifts the ratio between reduced and oxidized glutathione toward the oxidized state (Spoel and Loake, 2011). When SA and JA were applied simultaneously, the pattern of glutathione increase was the same as after treatment with SA alone, suggesting a role for redox regulation in prioritization of the SA pathway over the JA pathway (Koornneef et al., 2008a). So far, it is unclear how the SA-induced cellular reduction can influence JA-inducible responses.

Master regulator NPR1 is essential for SA/JA crosstalk and, therefore, the importance of SA-induced redox changes in SA/JA crosstalk could be related to reduction and translocation of NPR1 to the nucleus. However, the nuclear localization of NPR1 that follows SA-induced monomerization is, although essential for SA-responsive gene expression, not needed for SA-mediated suppression of JA-dependent genes (Spoel et al., 2003; Leon-Reyes et al., 2009). This was shown with *Arabidopsis* plants that overexpress a fusion protein of NPR1 that was retained in the cytosol: stimulation of the SA pathway in these plants resulted in a wild-type level of suppression of JA-induced *PDF1.2* (Spoel et al., 2003). The role of NPR1 in the cytoplasm for SA/JA crosstalk was confirmed in rice (*Oryza sativa*), where overexpression of OsNPR1 suppressed JA-responsive gene expression and defense against insects. However, when a mutated form of OsNPR1 was overexpressed that was constitutively present in the nucleus, herbivore resistance and expression of a JA-responsive gene were not affected (Yuan et al., 2007). Although NPR1 is exclusively needed in the cytosol for SA/JA crosstalk, it is still possible that redox-mediated modification of NPR1 is important in SA/JA crosstalk, for example if there is a role for the monomeric form of NPR1 in the cytosol to suppress JA signaling (Spoel et al., 2003; Beckers and Spoel, 2006). Alternatively, redox signaling may be important for post-translational modification of other factors with a role in SA/JA crosstalk, as described below.

The importance of redox regulation in SA/JA crosstalk is supported by the role of glutaredoxins (GRXs) in this phenomenon. GRXs are small ubiquitous redox enzymes that use glutathione to reduce their targets (Ndamukong et al., 2007; Ströher and Millar, 2012). SA is known to induce the expression of at least two GRXs, namely GRX480 and GRXS13, which are members of the group III class of GRXs in *Arabidopsis*. Overexpression of GRX480 blocks the induction of *PDF1.2* by JA, and overexpression of GRXS13 makes plants more susceptible to the necrotrophic fungus *Botrytis cinerea*, suggesting a role for both GRXs in suppression of JA signaling (Ndamukong et al., 2007; Camera et al., 2011). In fact, 10 more group III GRXs, which are also called ROXYs, are able to suppress activation of the *ORA59* promoter and are thus potentially involved in suppression of the JA pathway (Zander et al., 2012). Their antagonistic action on JA responses is likely downstream of NPR1, because expression of *GRX480* is reduced in the *npr1-1* mutant and overexpression of GRX480 in the *npr1-1* background still results in suppression of *PDF1.2* expression (Zander et al., 2012; Herrera-Vásquez et al., 2014). TGA transcription factors that are implicated in different hormonal signaling pathways and in SA/JA crosstalk (described more in-depth in SA-Inducible Expression of Transcription Factor Genes that Suppress JA Responses) are possible targets of group III GRXs, as they are shown to interact with each other (**Figure 1C**). Moreover, JA-induced *PDF1.2* expression is not impaired when GRX480 is overexpressed in the triple mutant *tga2/tga5/tga6* background, showing that the function of this GRX in suppression of JA-responses is dependent on these TGA transcription factors (Ndamukong et al., 2007; Zander et al., 2012).

### Sequestration and Degradation of Transcription Factors by SA

Salicylic acid could antagonize JA signaling by preventing accessibility of JA-responsive transcriptional regulators to their target genes. This could be achieved by sequestering transcription factors in inactive complexes or by degradation of positive regulators.

#### Sequestering Transcriptional Regulators by Complexation

By directing transcription factors to the cytosol, the possibility to activate transcription is obviously obstructed. In addition, transcription factors can be kept in check in the nuclear compartment as well, by inducing complex formation with other proteins that inhibit binding to the DNA, resulting in reduced transcription. There are no examples yet of SA-mediated sequestration of transcription factors leading to antagonism of JA signaling. However, some other plant hormone signaling interactions have been reported to be partly regulated via this mechanism, of which an example is the interaction between the SA and the ABA signaling pathways. The transcription factor WRKY40 is induced by SA and suppresses expression of the ABA-responsive genes *ABI4* and *ABI5*. After ABA treatment, the ABA receptor ABAR interacts with WRKY40, which is then recruited to the cytosol. By this recruitment, binding of WRKY40 to ABA responsive promoters is inhibited and repression of ABA responsive genes is lifted (Shang et al., 2010; Liu et al., 2012).

In animal cells, cytosolic sequestration of a transcriptional regulator was shown to control the antagonistic interaction between SA and prostaglandin signaling, which shares several aspects with SA/JA crosstalk in plants. SA and aspirin block the formation of prostaglandins in animal cells, which are considered structural analogs of JA in plants. SA induces retention of transcription factor NF-κB in the cytoplasm by enforcing its interaction with IκB. In response to stress, IκB kinase is activated and degrades IκB, leading to nuclear localization of NF-κB, which then activates gene expression, necessary for the production of prostaglandins. In cells that are exposed to SA, degradation of IκB is inhibited, which prevents the nuclear translocation of NF-κB. Interestingly, IκB in animals has structural similarity with NPR1 (reviewed by Spoel and Dong, 2012). In plants, the cytosolic location of NPR1 is important for SA-mediated antagonism of JA-responsive gene expression (Spoel et al., 2003; Stein et al., 2008). One possible function for cytosolic NPR1 is that it may sequester JA-regulated transcriptional activators in the cytoplasm, thereby preventing them from moving to the nucleus and activating transcription. However, whether SA can interfere with translocation of JA-responsive transcription factors to the nucleus remains to be demonstrated.

In the nucleus, transcription factors can be prevented from binding DNA and thus activating gene expression by interacting with repressor proteins, which have been reported to function as important regulators in several hormone signaling pathways (Robert-Seilaniantz et al., 2011). JAZ proteins in the JA pathway are examples of such repressors. JA-induced ubiquitination of JAZ proteins mediates their degradation via the 26S proteasome, which releases their repressive effect on positive transcriptional regulators. By increasing the stability of repressor proteins, hormones can antagonize another hormone’s action. An example of this crosstalk mechanism is found in the SA-auxin interaction. Parallel to JAZ repressor proteins in the JA pathway, AUX-IAA proteins are the negative regulators that bind and inactivate activators of auxin signaling. Binding of auxin to F-box proteins TIR1 and TIR1-related proteins, which act as auxin receptors, leads to degradation of AUX-IAA repressors. SA was shown to inhibit the auxin signaling pathway through stabilization of AUX/IAA repressor proteins, probably indirectly through repression of TIR1. In this way, SA could lift the disease promoting effect of auxin in the infection of *Arabidopsis* by *Pseudomonas syringae* (Wang et al., 2007). Also crosstalk between JA and GA pathways is regulated through interaction with their key repressor proteins, JAZs and DELLAs, respectively. In the absence of GA, stabilized DELLA can interact with JAZ proteins, thus reducing the repressive effect of JAZ on JA-responsive gene expression. DELLAs are degraded when GA levels rise, leading to enhanced suppression of JA signaling by JAZs (Hou et al., 2010; Pieterse et al., 2014). On the other hand, JA delays GA-mediated degradation of DELLAs, which is associated with a reduction in growth, suggesting that the trade-off between JA-dependent defense and GA-dependent growth can be regulated by the DELLA-JAZ signaling module (Yang et al., 2012). There is no evidence, however, that SA interferes with the stability of JAZs to antagonize JA signaling. First, JAZ1 and JAZ9, two of the most important JAZ proteins, are still degraded in JA-treated *Arabidopsis* when plants are additionally treated with SA. Second, SA was shown to antagonize the JA signaling pathway downstream of COI1, the F-box protein that interacts with JAZ repressor proteins to target them for ubiquitination (Van der Does et al., 2013).

#### SA-Mediated Degradation of JA-Regulated Transcription Factors

Salicylic acid-induced degradation of activating transcription factors of JA signaling could contribute to the repression of JA-responsive genes. Recently, SA was shown to lead to degradation of ORA59, a positive regulator in the ERF branch of the JA pathway. A whole-genome expression profiling analysis showed that the GCC-box was overrepresented in MeJA-induced genes that were antagonized by SA at 24 h after treatment with a combination of the hormones. The GCC-box was subsequently shown to be sufficient for suppression by SA (Van der Does et al., 2013). Similarly, the GCC-box was enriched in promoters of ethylene-induced genes that were suppressed by SA (Zander et al., 2014). The GCC-box is an essential promoter element for activation of *PDF1.2* expression and ERF transcription factor ORA59 is an important regulator in this activation (Zarei et al., 2011). Van der Does et al. (2013) suggested that downregulation of transcription of *ORA59* is not essential for SA/JA crosstalk, but showed that protein levels of ORA59 diminished after SA treatment, suggesting that SA could target positive regulators in the JA pathway for degradation. So far, degradation of other positive regulators of JA signaling has not been reported. The degradation rate of MYC2, master regulator of the MYC branch in the JA pathway, is likely not influenced by SA (Chico et al., 2014).

### Phosphorylation of Transcription Factors Influences Transcription

Perception of pathogenic microbes by the plant leads to activation of mitogen-activated protein kinases (MPKs) that can subsequently phosphorylate transcriptional regulators. Phosphorylation of transcription factors influences gene transcription by changing the binding strength to DNA, or affecting sequestration or stability (Tena et al., 2011). In particular MPK3, MPK4, and MPK6, which act at the last step of MAPK signaling cascades, are known to phosphorylate transcription factors and have been implicated in immune signaling (Meng and Zhang, 2013). For example, phosphorylation of WRKY33 by MPK3 and MPK6 is likely responsible for the WRKY33-mediated induction of the *WRKY33* gene itself and of *PAD3*, which is a camalexin biosynthesis gene (Mao et al., 2011). It has also been suggested that WRKY33 is controlled by sequestration in a complex with MKS1 and MPK4. Upon bacterial pathogen attack the activated MAPK signaling cascade phosphorylates MKS1, which leads to disassociation from MPK4 so that WRKY33 is released from the complex and could bind to the promoter of *PAD3* (Qiu et al., 2008).

There is not much known about the role of MAPK cascades in the interplay between different hormone pathways. MAPK cascades are important in the JA pathway, so inhibition of MAPK cascades by SA could be an effective way to antagonize JA signaling. For example, JA activates MPK6 and many AP2/ERFs transcription factors are phosphorylated and activated by MPK6, among which positive regulators ERF6 and ERF104 (Takahashi et al., 2007; Bethke et al., 2009; Popescu et al., 2009; Meng et al., 2013). It is not known if SA can prevent this phosphorylation to inhibit activation of the JA-regulated AP2/ERF transcription factors. MPK4 was thought to function as an integrator of SA and JA signaling as the mutant *mpk4* constitutively expresses SA-inducible *PR* genes and fails to express *PDF1.2*, which correlates with enhanced resistance to biotrophic pathogens and increased susceptibility to necrotrophic pathogens (Petersen et al., 2000; Brodersen et al., 2006). However, recently it was suggested that MPK4 is guarded by the R protein SUMM2. Reduction of the kinase activity of MPK4 by the bacterial effector HopAI1 is monitored by SUMM2, and leads to activation of SA-dependent defense responses (Zhang et al., 2012b). The effects of MPK4 on SA signaling are thus indirect, and this makes a role for MPK4 as an integrator of SA and JA signaling unlikely. However, whether MPK4’s role in JA signaling is a direct or indirect one needs to be studied further.

### SA-Inducible Expression of Transcription Factor Genes that Suppress JA Responses

Salicylic acid may also antagonize JA-inducible gene transcription by inducing the expression of genes encoding transcriptional regulators that interfere with JA signaling. These SA-induced regulators could inhibit a positive regulator of JA-inducible gene expression by interacting with it, as is described for the GRX480-TGA interaction in “Role of Reduction of Transcriptional Regulators in SA Signaling.” Alternatively, SA could induce transcription of suppressive transcription factors that directly bind to the promoter of JA responsive genes to repress their expression. Examples of TGA, ERF, WRKY, and bHLH transcription factors that are induced by SA and inhibit JA-dependent transcription are reviewed below.

#### TGA Transcription Factor Family

TGA transcription factors have a role in various hormone-regulated transcriptional responses. They can generally activate SA-dependent gene expression, but are also known to have both positive and negative effects on JA/ethylene-dependent responses. TGA transcription factors are a class of bZIP transcription factors that bind to the *as-1* element (TGACG) in promoters. In *Arabidopsis*, 10 TGAs exist of which several have been shown to interact with NPR1 (reviewed by Gatz, 2013). The *PR1* promoter contains an *as-1* element, and the triple mutant *tga2/tga5/tga6* is, like *npr1*, compromised in SAR and does not express *PR1* upon treatment with the SA-mimic INA (Zhang et al., 2003). In response to SA, a ternary complex of TGA, NPR1, and DNA is formed that can activate transcription of *PR1* (**Figure 1A**). In non-induced conditions, suppression of *PR1* by TGAs has also been reported (Rochon et al., 2006; Pape et al., 2010). TGAs are important for activation of JA/ethylene-dependent genes as well. Although mutant *tga2/tga5/tga6* adult plants responded with *PDF1.2* induction upon treatment with JA, they did not express *PDF1.2* in response to ethylene or *B. cinerea* infection (Zander et al., 2010).

In addition, TGAs can be essential for suppression of JA responsive genes by SA, as JA-induced *PDF1.2* is not suppressed after a combination treatment with SA in mutant *tga2/tga3/tga5/tga6* (Leon-Reyes et al., 2010a). Microarray analysis comparing wild-type and *tga2/tga5/tga6* mutant plants showed that after treatment with ethylene precursor ACC, 374 genes were induced in wild-type plants, of which 136 were dependent on TGA2/TGA5/TGA6. Half of these ACC-inducible TGA-dependent genes were, in wild-type plants, suppressed by SA after a combination treatment of ACC with SA. This suggests a role for TGAs in both activation of ethylene-responsive genes and SA-mediated repression of these genes (Zander et al., 2014). The *PDF1.2* promoter contains an *as-1* element, but this was shown not to be important for the antagonistic effect on JA-induced *PDF1.2* expression by SA (Spoel et al., 2003). However, Zander et al. (2014) showed that the TGAs directly target the *as-1* element in the promoter of *ORA59* and could regulate both induction of *ORA59* by ACC treatment and suppression of *ORA59* by SA (**Figure 1C**). Transcriptional regulation of *ORA59* by TGAs is in line with the observation that the GCC-box is enriched in the promoter elements of ACC-induced, SA-suppressed genes. How can TGA factors act as both activators and repressors in different hormone signaling pathways? Possibly, different co-factors can be recruited to TGA factors depending on both the promoter context and the hormonal context. In the case of activation of transcription by SA, TGAs have been shown to interact with transcriptional activators NPR1 and GRAS protein SLC14 (Rochon et al., 2006; Fode et al., 2008). Upon JA accumulation, TGAs may interact with so-far unknown JA signaling regulators to promote JA responsive gene expression. When SA/JA crosstalk is activated, SA induces GRXs, which could interact with TGAs on the *ORA59* promoter leading to repression of JA-inducible genes (**Figure 1C**). GRXs were shown to down-regulate *ORA59* expression in a TGA-dependent manner, as discussed in “Role of Reduction of Transcriptional Regulators in SA Signaling” (Zander et al., 2012).

Both Zander et al. (2014) and Van der Does et al. (2013) point to ORA59 as a major target of antagonism by SA. However, while the first show that SA targets expression of *ORA59*, the protein levels of ORA59 were shown to be influenced by SA by the latter. The apparent discrepancy between these two studies could partly be explained by the different combination of hormones that both groups studied, SA-ethylene or SA–JA, respectively. Support for differences in crosstalk mechanisms depending on hormonal context comes from the observation that in an ethylene-rich environment the SA-antagonized expression of JA-inducible *PDF1.2* became independent of NPR1 (Leon-Reyes et al., 2009) or was even completely impaired when plant tissue was exposed to high levels of ethylene prior to treatment with SA (Leon-Reyes et al., 2010a). However, it is very well possible that ORA59 is regulated by SA at both the transcriptional and post-translational level, and that both mechanisms complement each other (**Figure 1C**).

#### ERF Transcription Factor Family

Transcription factors of the ERF subfamily of AP2/ERF family of transcription factors can bind the GCC-box and can act as activators, such as ORA59, but also as repressors of transcription. Fourteen of the 122 ERFs in *Arabidopsis* contain an EAR domain, which is an active repressor domain that interacts with the general co-repressor TPL (Nakano et al., 2006). EAR-domain-containing ERF4 and ERF9 were shown to be able to suppress *PDF1.2* expression (McGrath et al., 2005; Maruyama et al., 2009). Because of the importance of the GCC-box in SA/JA crosstalk, the suppression of JA-responsive genes may, besides through negative regulation of ORA59 by SA (covered in SA-Mediated Degradation of JA-Regulated Transcription Factors and TGA Transcription Factor Family), in part be regulated by suppressive SA-induced ERFs. This hypothesis has up to now not been tested.

#### WRKY Transcription Factor Family

WRKY transcription factors are foremost known for their inducibility by SA and pathogens, and their role in regulating SA-dependent gene expression. There are, however, also examples of WRKYs that positively regulate other hormone-regulated genes, including JA-responsive defense genes (Journot-Catalino et al., 2006; Xu et al., 2006). The W-box (C/TTGACC/T) is a DNA element that is bound by WRKY transcription factors (Eulgem and Somssich, 2007). Importantly, the W-box motif was reported to be enriched in JA-responsive genes that were antagonized by SA (Van der Does et al., 2013), suggesting the involvement of WRKYs in SA/JA crosstalk as well. Indeed, several WRKYs have been implicated in suppression of JA-induced *PDF1.2* expression (**Figure 1C**). Overexpression of SA-induced WRKY70 suppressed MeJA-induced *PDF1.2* expression (Li et al., 2004, 2006). However, in a *wrky70* mutant, JA-dependent genes were induced by JA and suppressed by the combination treatment, indicating that WRKY70 is sufficient but not required for SA/JA crosstalk (Ren et al., 2008; Leon-Reyes et al., 2010a). Redundancy of different WRKYs could possibly explain the lack of a crosstalk phenotype of the single *wrky70* mutant, as double and triple mutants of *wrky70* with *wrky46* and *wrky53* did show enhanced *PDF1.2* expression after MeJA treatment (Hu et al., 2012). Overexpression of the transcription factor MYB44 also led to suppression of the JA marker genes *VSP1* and *PDF1.2*, which is likely established through activation of *WRKY70*. MYB44 is inducible by SA and binds to the *WRKY70* promoter leading to its expression (Shim et al., 2013; Zou et al., 2013). Furthermore, WRKY62 was suggested to function in suppression of JA responses, because a *wrky62* mutant displayed enhanced expression of JA responsive genes, while an overexpressor exhibited reduced expression. *WRKY62* is induced by SA and was suggested to act downstream of cytosolic NPR1 (Mao et al., 2007). To end, WRKY41 has been implicated in suppression of JA responsiveness, since overexpression of WRKY41 led to increased *PR5* and reduced *PDF1.2* expression. However, in contrast to the aforementioned *WRKY* genes, *WRKY41* is likely not a direct target of NPR1 and SA only slightly induces *WRKY41* expression (Higashi et al., 2008).

Studies on the *ssi2* mutant revealed two other WRKYs that are involved in SA/JA crosstalk. The *ssi2* mutant was initially identified in a screen for *npr1* suppressors and displays high SA responses while JA responses are repressed (Shah et al., 2001). The increased SA levels were not needed for the repression of JA responses, but instead lowered levels of 18:1 fatty acids appeared to regulate the repression of JA signaling (Kachroo et al., 2001, 2003; Nandi et al., 2005). In *ssi2* mutants, 19 *WRKYs* were induced, of which five in a SA-independent manner. Double mutants of *ssi2* with *wrky50* or *wrky51* restored the induction of *PDF1.2* and resistance against *B. cinerea* without altering the 18:1 fatty acid levels. WRKY50 and WRKY51 thus negatively regulate JA responses under low 18:1 conditions. Single and double mutants of *wrky50* and *wrky51* also failed to suppress *PDF1.2* and *VSP2* after a combination treatment with SA and JA (Gao et al., 2011). Therefore, these two WRKYs seem to play important roles in the suppression of JA responses.

How can WRKY transcription factors repress JA responses? After their induction by SA, they could bind to W-boxes in JA-responsive genes to inhibit their expression directly or indirectly (Van der Does et al., 2013). There is no experimental proof of this repressive mechanism under the influence of SA yet, but recently WRKY51 has been reported to interact with JAV1, a VQ-motif containing protein that negatively regulates JA responses and acts in the nucleus (Hu et al., 2013).

#### bHLH Transcription Factor Family

Transcription factors of the bHLH family, including MYC2, play crucial roles in the JA signaling pathway. MYC2 is a master regulator of JA responses (reviewed by Kazan and Manners, 2013). The last 2 years have witnessed an boost in bHLHs that function as negative regulators in the JA signaling pathway (Nakata et al., 2013; Sasaki-Sekimoto et al., 2013; Song et al., 2013; Fonseca et al., 2014). Whether these repressive bHLHs are manipulated by SA to establish SA/JA crosstalk is currently unknown, but they are not obviously regulated at the transcription level by SA (BAR public database).

### SA/JA Crosstalk could be Enforced by Chromatin Modification at Target Genes

Salicylic acid can further control gene expression by remodeling of chromatin around target genes. Chromatin is the complex of DNA and histones and its condensed structure can reduce accessibility of DNA and thus inhibit transcription. Modifications of chromatin can result in local loosening of this structure, which creates access for transcriptional machinery and regulatory proteins to the DNA. Chromatin modifications include methylation, acetylation, phosphorylation, ubiquitination, or sumoylation of histones (Iwasaki and Paszkowski, 2014). Acetylation of histones is associated with activation of genes, while deacetylation of histones is correlated with gene repression. Enzymes called histone acyltransferases and histone deacetylases (HDA) can carry out these respective histone modifications (Liu et al., 2014). HDA6 and HDA19 were described to interfere with JA signaling. HDA6 interacts with JAZ1, JAZ3, and JAZ9 and is recruited to repress EIN3/EIL1-dependent transcription (Zhu et al., 2011). In contrast, HDA19 was reported to have a positive role in the ERF branch and in defense against *Alternaria brassicicola* (Zhou et al., 2005). HDA19 also targets SA signaling by binding to the *PR1* and *PR2* promoters leading to their repression (Choi et al., 2012), and by reducing transcriptional activity of WRKY38 and WRKY62 (Kim et al., 2008). Since chromatin remodeling plays an important role in SA and JA signaling, it could also well be manipulated by SA to antagonize JA signaling. However, Koornneef et al. (2008b) showed that at the *PDF1.2* promoter there was no change in acetylation of histones after exogenous application of a combination of SA and MeJA.

Chromatin modifications are also described to be an important mechanism to prime plants for enhanced defense (Conrath, 2011). Interestingly, it was suggested that priming and SA/JA crosstalk could be carried over to offspring through acetylation and methylation of histones as well. Luna et al. (2012) showed that *Arabidopsis* plants that were inoculated with the bacterial pathogen *P. syringae* in the first generation, were more resistant to *P. syringae* and the oomycete pathogen *Hyaloperonospora arabidopsidis* in the next generation, and more susceptible to the necrotrophic pathogen *A. brassicicola*. This correlated with increased *PR1* expression and reduced *VSP2* and *PDF1.2* expression in the second generation and was dependent on NPR1. Acetylation of histone H3 at Lys-9 (H3K9) at the *PR1* promoter, which is associated with increased transcription, was enhanced in these plants. Conversely, tri-methylation of H3K27, which is associated with transcriptional silencing, was enriched at the *PDF1.2* promoter (**Figure 1C**), suggesting that histone modifications were responsible for the observed increased or decreased transcription (Luna et al., 2012). It is not clear yet how these changes can be transmitted to offspring, since there is no evidence that histone modifications are inherited. DNA methylation, which is often associated with histone modifications, is a possible modification that could be passed on to next generations. DNA methylation was shown to have an effect on SA- and JA-regulated responses: epiRIL lines, which are identical at the DNA sequence level but highly variable at the level of DNA methylation, showed differences in responsiveness to both treatments (Latzel et al., 2012).

## Rewiring of Hormone-Regulated Transcriptional by Pathogens

In the evolutionary arms race, pathogens have evolved effectors that are secreted into plant cells upon infection to reduce disease resistance or increase plant susceptibility (reviewed by Kazan and Lyons, 2014). Interestingly, several pathogen effectors can highjack a plant’s intricate hormonal crosstalk mechanism for their own good, resulting in lower induction of effective defenses. Some effectors are hormones themselves or are hormone-mimics that disturb the hormone balance in plants. The most famous example of such an effector is the JA-mimic coronatine, that is secreted by *Pseudomonas* pathogens and suppresses SA signaling (Zheng et al., 2012). More recently, effectors that interfere with signaling hubs in transcriptional regulation of JA signaling, such as JAZs, have been discovered. Effectors HopZ1a and HopX1 of two different *Pseudomonas* pathogen strains bind to and degrade JAZ repressor proteins, leading to activation of JA signaling and concomitant suppression of SA-regulated defense signaling (Jiang et al., 2013; Gimenez-Ibanez et al., 2014).

Other effectors can establish antagonism of SA signaling by manipulating the plant transcriptional machinery via interference with Mediator subunits. Mediator is a multi-protein transcriptional co-activator complex, which functions as a bridge between transcription factors and RNA polymerase II. Mediator recruits RNA polymerase II to promoters in response to different signals and controls the polymerase activity during transcription initiation and elongation (Conaway and Conaway, 2011). Several Mediator subunits have been implicated in SA- and/or JA-dependent gene expression. Mediator subunit MED16 was shown was shown to be important in defense against both biotrophic and necrotrophic pathogens by regulating SA- and JA/ethylene-responsive transcription and could therefore be viewed as a node of convergence between SA- and JA/ethylene-dependent pathways (Wathugala et al., 2012; Zhang et al., 2012a). Subunit MED25 was shown to be important for activation of JA-dependent genes, and likely acts through interaction with JA-responsive transcription factors, including ERF1, ORA59, and MYC2 (Çevik et al., 2012). The subunit MED19 positively regulates SA-dependent resistance that is effective against *H. arabidopsidis.* MED19 was shown to be targeted for degradation by the *H. arabidopsidis* effector HaRxL44. Expression of HaRxL44 in plants led to induction of JA-responsive genes, a response that is observed in *med19* plants as well (Caillaud et al., 2013). These data suggest that HaRxL44 induces degradation of MED19 to rewire transcription from SA-responsive to JA-responsive, leading to enhanced infection by *H. arabidopsidis*. This example illustrates the highly sophisticated manner in which effectors manipulate the plant transcriptional machinery to influence hormonal signaling.

## Conclusion and Perspectives

In the last years, knowledge on the interplay between different plant hormone signaling pathways has vastly increased. In this review we focused on the molecular mechanisms (potentially) underlying antagonistic effects of SA on JA-mediated transcriptional responses and highlighted several transcriptional regulators (like NPR1, TGA, WRKY, and ORA59) as signal integrators. However, there is still much unknown about hormonal crosstalk mechanisms. The use of whole-transcriptome sequencing techniques after combinatorial hormone treatment or pathogen infection will aid in the identification and characterization of additional transcriptional regulators that can act as nodes of convergence in multiple signaling pathways (Van Verk et al., 2013). Combining transcriptome data with ChIP-seq or DNase-seq studies, which can identify DNA sites occupied by transcription factors, can provide more detailed knowledge on the mechanisms by which these crosstalk transcriptional regulators rewire hormonal signaling. In addition, more intensive proteomic studies are necessary to get a full scale picture of the posttranslational modifications that influence the action of key transcriptional regulators. The knowledge gained from pharmacological experiments, in which combinations of hormones are applied exogenously, should be corroborated under biological conditions that trigger hormonal crosstalk, like (combinatorial) pathogen infection. Insights into the crosstalk signaling hubs that function in complex hormonal signaling networks will not only increase our fundamental knowledge on plant immune signaling but can also provide leads to develop crops with multi-attacker resistance and optimal growth.

## Conflict of Interest Statement

The authors declare that the research was conducted in the absence of any commercial or financial relationships that could be construed as a potential conflict of interest.
